# Modelling the Relative Vaccine Efficacy of ARCT-154, a Self-Amplifying mRNA COVID-19 Vaccine, versus BNT162b2 Using Immunogenicity Data

**DOI:** 10.3390/vaccines12101161

**Published:** 2024-10-11

**Authors:** Van Hung Nguyen, Pascal Crépey, Jean Marie Pivette, Ethan Settembre, Sankarasubramanian Rajaram, John Youhanna, Aimee Ferraro, Cheng Chang, Josephine van Boxmeer, Joaquin F. Mould-Quevedo

**Affiliations:** 1 VHN Consulting, 69370 Lyon, France; 2 RSMS—Inserm U 1309, Arènes—UMR 6051, EHESP, CNRS, IEP Rennes, University of Rennes, 35043 Rennes, France; 3 VHN Consulting, 22450 Trezeny, France; 4 CSL Seqirus, Waltham, MA 02451, USA; 5 CSL Seqirus, Maidenhead SL6 8AA, UK; 6 CSL Seqirus, 1105 BJ Amsterdam, The Netherlands; 7 CSL Seqirus, Summit, NJ 07901, USA

**Keywords:** ARCT-154, BNT162b2, self-amplifying mRNA vaccine, older adults, efficacy

## Abstract

**Background**: Self-amplifying mRNA vaccines have the potential to increase the magnitude and duration of protection against COVID-19 by boosting neutralizing antibody titers and cellular responses. **Methods**: In this study, we used the immunogenicity data from a phase 3 randomized trial comparing the immunogenicity of ARCT-154, a self-amplifying mRNA COVID-19 vaccine, with BNT162b2 mRNA COVID-19 vaccine to estimate the relative vaccine efficacy (rVE) of the two vaccines over time in younger (<60 years) and older (≥60 years) adults. **Results**: By day 181 post-vaccination, the rVE against symptomatic and severe Wuhan-Hu-1 disease was 9.2–11.0% and 1.2–1.5%, respectively, across age groups whereas the rVE against symptomatic and severe Omicron BA.4/5 disease was 26.8–48.0% and 5.2–9.3%, respectively, across age groups. Sensitivity analysis showed that varying the threshold titer for 50% protection against severe disease up to 10% of convalescent sera revealed incremental benefits of ARCT-154 over BNT162b2, with an rVE of up to 28.0% against Omicron BA.4/5 in adults aged ≥60 year. **Conclusions**: Overall, the results of this study indicate that ARCT-154 elicits broader and more durable immunogenicity against SARS-CoV-2, translating to enhanced disease protection, particularly for older adults against Omicron BA.4/5.

## 1. Introduction

The COVID-19 pandemic resulted in approximately 6.9 million deaths and 677 million cases reported worldwide between 2020 and 2023 and continues to be a leading cause of respiratory infection hospitalizations. In the US, there have been over 600,000 hospitalizations and 50,000 deaths due to COVID-19 reported since September 2023, with 17% of hospitalized patients requiring intensive care unit (ICU) admission and 7% requiring mechanical ventilation [1]. Similarly, in the UK, nearly 95,000 patients have been hospitalized for COVID-19 during the same time period, with 8,500 deaths, indicating the continued need for preventative measures to reduce the disease burden [2].

Although vaccination against COVID-19 has been a critical tool for reducing the incidence of severe outcomes and death, waning protection has been observed for the original vaccines over time and with the emergence of new SARS-CoV-2 variants and subvariants [3]. Given this reduced effectiveness, COVID-19 vaccines recommended since May 2023 contained an updated formulation targeting the spike protein of the Omicron variant XBB.1.5, with the aim of providing enhanced protection against circulating variants compared to the original formulation [4].

Despite being relatively unknown prior to the COVID-19 pandemic, mRNA vaccine technology allowed rapid development of candidate vaccines, resulting in the first vaccines being available within a year of the identification and sequencing of SARS-CoV-2 [5,6]. Advantages of the mRNA platform over more traditional vaccine platforms include rapid development, high efficacy, and the potential to easily update formulations as required. However, standard mRNA vaccines have some limitations, including the need for multiple booster doses to maintain protection [7,8]. To address these challenges, a next generation of mRNA vaccines based on self-amplifying (sa) mRNA is under development, which has the potential to elicit both stronger and more durable immune responses and provide broader protection against emerging variants [9].

In conventional mRNA vaccines such as BNT162b2, each mRNA molecule contains a region encoding the antigen of interest flanked by 5′ and 3′ untranslated regions, together with a cap and poly (A) tail, to resemble naturally occurring mRNA in mammalian cells [7]. In contrast, sa-mRNA vaccines additionally contain replication machinery such as genes encoding viral replicase that first make more mRNA copies of the gene of interest. Each of the mRNA copies is then translated into protein, increasing protein production compared with conventional mRNA vaccines and thereby potentially enhancing the immune response [9]. As this self-amplification results in increased antigen levels within the cell, sa-mRNA vaccines are therefore expected to be dose-sparing compared to conventional mRNA vaccines, with less injected mRNA required for an equivalent immune response.

ARCT-154 is a lipid nanoparticle sa-mRNA vaccine containing mRNA from the Venezuelan equine encephalitis virus (VEEV) genome, with the structural genes replaced by the D614G variant of the full-length SARS-CoV-2 spike protein [10]. ARCT-154 has been evaluated as a primary series in a pooled phase 1/2/3a/3b study in COVID-19 vaccine-naïve patients in Vietnam [11]. In the study, 94.1% (95% confidence interval [CI]: 92.1–95.8) of patients seroconverted, with a mean geometric mean-fold rise from a baseline of 14.5 (95% CI 13.6–15.5). The efficacy of the primary series was estimated at 56.6% (48.7–63.3) against any COVID-19, and 95.3% (80.5–98.9) against severe COVID-19, with similar estimates for patients <60 and ≥60 years of age. ARCT-154 has also been evaluated as a booster in a phase 3 trial in healthy adults in Japan (jRCT registry no. jRCT2071220080). Interim data published after 6 months of follow-up demonstrated non-inferior immunogenicity to BNT162b2 against the Wuhan-Hu-1 virus and superior immunogenicity against the Omicron BA.4/5 variant [12]. The efficacy of ARCT-154 as a booster dose in individuals who have already received prior doses of COVID-19 vaccines has not yet been evaluated. Based on the immunogenicity findings from the phase 3 study comparing a booster dose of BNT162b2 and ARCT-154, we hypothesized that the efficacy of the two vaccines would also be similar or higher for ARCT-154 compared to BNT162. To this end, this study modelled the relative vaccine efficacy (rVE) of ARCT-154 versus BNT162b2 in adults <60 years and ≥60 years of age for 6 months post-vaccination, using immunogenicity data from the phase 3 booster study.

## 2. Methods

### 2.1. Phase 3 Study Design

Input parameters for the estimation of rVE in the model were taken from a randomized double-blind active-controlled comparative study of ARCT-154 in healthy adults conducted in Japan between December 2022 and February 2023. Full details of the study design, participant eligibility, and objectives have been published previously [12]. In brief, individuals ≥18 years of age who had previously received two documented doses of mRNA-1273 or BNT162b2 vaccines and a third dose of BNT162b2 at least 3 months previously were eligible for inclusion (*N* = 828). Participants received a single dose of ARCT-154 or BNT162b2 on day 1 of the study. Immunogenicity was evaluated in terms of geometric mean titers (GMTs) of neutralizing antibodies against Wuhan-Hu-1 and Omicron BA.4/5 sublineage SARS-CoV-2 pseudoviruses on days 1, 29, 91, and 181. The geometric mean titer (GMT) is a statistical measure used in immunology to describe the central tendency of antibody levels within a group. It is calculated by taking the logarithm of all individual titers, computing the arithmetic mean of these values, and then taking the antilogarithm of the resulting mean. Immunogenicity was evaluated for the overall study population, as well as stratified by age group (<60 years and ≥60 years). Analysis was performed on the per-protocol subset 1 (PPS-1), which included randomized participants who received the study vaccine, had no major protocol violations, and were seronegative for the SARS-CoV-2 nucleocapsid before study vaccine administration, indicating no recent prior infection (see [12] for further details).

### 2.2. Modelling the Relationship between Neutralizing Antibody Titers and Efficacy against SARS-CoV-2 Infection

We used the same methodology as previously described by Khoury et al. [13] to estimate the relationship between neutralizing antibody levels and protection from symptomatic and severe SARS-CoV-2 infection. In brief, Khoury et al. [13] developed a logistic regression model to estimate the correlation between mean neutralizing antibody level (defined as the ratio of vaccine-induced GMTs to convalescent sera titers within the same study) and vaccine efficacy. Data for the model were derived from published studies that included convalescent sera and neutralizing antibody titers from seven of the available COVID-19 vaccines. Because the assays used varied across the studies, neutralizing antibody titers were normalized to the mean convalescent titer estimated using the same assay within each study. In our analysis, we meticulously reproduced the correlate of protection using R (version 4.4.0) to ensure accurate implementation of the data from the seven-study dataset previously used by Khoury et al. [13]. We retained the neutralization level required for 50% protection (N50) against detectable SARS-CoV-2 infection at 20.2% of the mean convalescent level, as estimated by Khoury et al. [13]. For the baseline scenario, we also retained the N50 against severe disease of 3% of the mean convalescent titer.

To estimate the rVE of ARCT-154 vs BNT162 against symptomatic and severe COVID-19, we used GMTs from days 29, 91, and 181 for <60-year olds and ≥60-year olds as input parameters for the model. As the phase 3 study of ARCT-154 did not include any convalescent immunity data, we assumed that the ratio of neutralization levels of the BNT162b2 vaccine to convalescent sera titers would be the same as that described in the Khoury et al. study (i.e., 2.37) [13]. Based on this, we then generated an assumed value for a theoretical convalescent sera titer for each time point per age group and applied this to predict a ratio of ARCT-154-induced GMTs to convalescent sera titer for inclusion in the model. This was then plotted using the model curve to estimate a value of vaccine efficacy. Relative vaccine efficacy (rVE) was calculated as the difference between the vaccine efficacy estimates for the two vaccines, expressed as rVE = (VE1 − VE2)/VE2, where VE1 was the vaccine efficacy of ARCT-154 and VE2 was the vaccine efficacy of BNT162b2.

### 2.3. Sensitivity Analysis: Severe COVID-19 Disease

Based on the model by Khoury et al. [13], the baseline N50 level against severe COVID-19 disease was assumed to be 3%, meaning a GMT of 3% of the convalescent sera titer. Because vaccines may not be as effective against currently circulating strains as estimated from the clinical trial data included in the original Khoury model, we also performed a sensitivity analysis by varying the N50 threshold up to 10%. All modelling and analyses were performed using R (version 4.4.0).

## 3. Results

### 3.1. Immunogenicity of ARCT-154 and BNT162b2 by Age Group in the Phase 3 Trial

In total, 828 participants were enrolled in the phase 3 study (ARCT-154: *n* = 420; BNT162b2: *n* = 408), of whom 759 were included in PPS-1 (i.e., had no evidence of recent SARS-CoV-2 infection). In both vaccine groups, GMTs against Wuhan-Hu-1 and Omicron BA.4/5 increased post-vaccination, with no clear differences in response between younger (<60 years) and older (≥60 years) adults. Point estimates of GMTs were generally highest on day 29 in the BNT162b2 group and on day 91 in the ARCT-154 group, with numerically higher responses against Wuhan-Hu-1 than Omicron BA.4/5 (Table 1).

### 3.2. Vaccine Efficacy and rVE against Symptomatic COVID-19 Disease

Using the ratio of neutralizing antibody titers to convalescent sera titers of the BNT162b2 vaccine as described in Khoury et al. [13] (i.e., 2.37), theoretical values for convalescent sera titers were estimated against Wuhan-Hu-1 and Omicron BA.4/5 for each time point and were used to estimate the relationship between neutralization level and vaccine efficacy. Estimated vaccine efficacy was highest against Wuhan-Hu-1 on day 29 and ranged from 94.3% to 95.1% and from 91.7% to 92.7% across age groups for ARCT-154 and BNT16b2, respectively. The waning of efficacy was greater for BNT162b2 than ARCT-154, with efficacy dropping to 85.6% in participants <60 years and 83.7% in participants ≥60 years by day 181, compared to 93.4% and 93.1%, respectively, for the ARCT-154 group. Similar trends were seen against Omicron BA.4/5, with the efficacy of BNT162b2 waning from 83.8% to 60.9% in participants <60 years and from 80.5% to 53.9% in those ≥60 years, compared with from 87.3% to 77.2% and from 84.2% to 79.7%, respectively, for ARCT-152.

**Figure 1:**
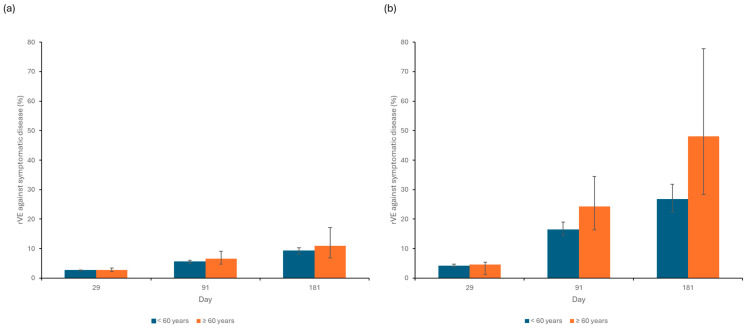
rVE of ARCT-154 vs. BNT162b2 against symptomatic COVID-19 caused by (**a**) Wuhan-Hu-1 and (**b**) Omicron BA.4/5 on days 29, 91, and 181 post-vaccination, by age group. rVE, relative vaccine efficacy.

The rVE of ARCT-154 vs BNT162b2 against symptomatic disease was estimated at 2.6% (95% CI: 2.4–2.7) and 2.8% (2.2–3.5) on day 29 against Wuhan-Hu-1 in participants <60 years and ≥60 years, respectively, rising to 9.2% (8.2–10.3) and 11.0% (6.6–17.1), respectively, by day 181. Against Omicron BA.4/5, the rVE ranged from 4.2% (3.8–4.7) and 4.6% (1.2–5.5) on day 29 to 28.6% (22.5–31.8) and 48.0% (28.4–77.8) by day 181, for the two age groups, respectively (Figure 1).

### 3.3. Vaccine Efficacy and rVE against Severe COVID-19 Disease

Efficacy against severe disease (based on the baseline scenario N50 of 3%) was predicted to be high for both vaccines against the Wuhan-Hu-1 strain, remaining above 97.5% through day 181 in both age groups. Against Omicron BA.4/5, vaccine efficacy ranged from 97.1% to 98.4% across age groups and vaccines on day 29. By day 181, the efficacy was between 96.4 and 97.0% for ARCT-154 and between 88.8% and 91.6% by day 181 across age groups.

**Figure 2:**
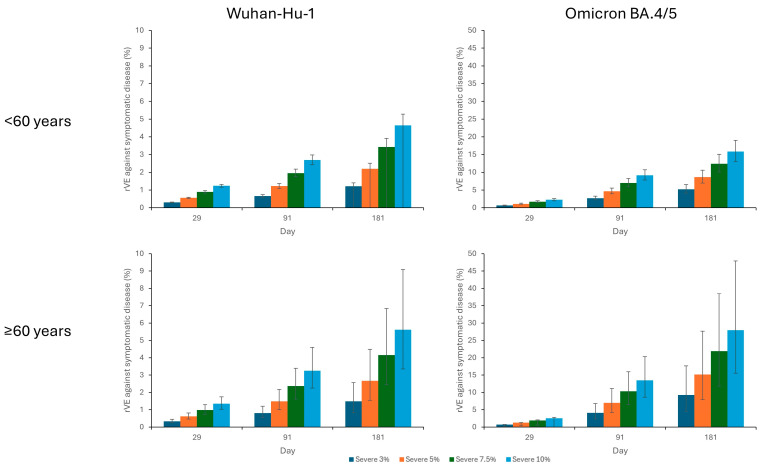
rVE of ARCT-154 vs BNT162b2 against severe COVID-19 on days 29, 91, and 181 post-vaccination, by age group, including sensitivity analysis varying the threshold titer considered protective against 50% of severe disease from 3% to 10% of convalescent sera. rVE, relative vaccine efficacy.

The rVE against severe disease caused by Wuhan-Hu-1 was low in both age groups, ranging from 0.3% (0.3–0.3) and 0.3% (0.3–0.5) on day 29 to 1.2% (0.0–1.4) and 1.5% (0.8–2.6) for participants <60 years and ≥60 years, respectively. The rVE against Omicron BA.4/5 was higher, with estimates ranging from 0.6% (0.5–0.7) and 0.7% (0.2–0.8) on day 29 to 5.2% (4.2–6.5) and 9.3% (4.6–17.7) by day 181 for participants <60 years and ≥60 years, respectively (Figure 2).

### 3.4. Sensitivity Analysis: rVE against Severe COVID-19

Sensitivity analysis varying the threshold titer considered protective against 50% of severe disease up to 10% of the convalescent sera titer showed incremental benefits of ARCT-154 over BNT162b2 with time. The greatest differences between vaccines were seen against Omicron BA.4/5 by day 181 in adults ≥60 years, with the rVE varying between 15.2% (7.9–27.7) and 28.0% (15.5–47.9) for thresholds titers of 5–10% of convalescent sera (Figure 2).

## 4. Discussion

Our modelling study indicates that based on neutralizing antibody titers, the sa-mRNA vaccine ARCT-154 is expected to have broader and longer-term efficacy compared to BNT162b2, including against future circulating Omicron variants. Differences between the two vaccines were greatest at six months post-vaccination in adults ≥60 years of age, particularly against Omicron BA.4/5, where the rVE against symptomatic infection was over 48%. Although both vaccines are expected to be highly efficacious against severe disease based on the 3% convalescent sera titer threshold, using a stricter definition of up to 10% of convalescent sera titers resulted in incremental benefits of ARCT-154 over BNT162b2, with an rVE of up to 28% six months post-vaccination in older adults. Although we did not directly estimate the effects on clinical outcomes, it is likely that these rVE estimates would correspond to greater clinical benefits in terms of reduced symptomatic and severe cases from the use of ARCT-154 compared with BNT162b2, particularly in older adults and against Omicron variants. The results of our analysis also support the hypothesis that sa-mRNA vaccines would be dose-sparing, as a single 0.5mL dose of ARCT-154 used in the phase 3 study containing 5 µg mRNA resulted in higher titers than the 30 µg mRNA included in the 0.3mL dose of BNT162b2 [12,14].

Both antibodies and cellular responses play key roles in the prevention of COVID-19 disease and severe outcomes. Neutralizing and non-neutralizing antibodies have multiple mechanisms of action in preventing COVID-19 from infecting cells by blocking binding of the spike protein with ACE2 receptors and co-receptors, by preventing conformational changes required for viral cell entry, and by the binding of infected cells to induce phagocytosis or targeted cell death [15]. Higher COVID-19 neutralizing antibody titers have been associated with a decreased likelihood of severe disease and increased survival, as well as a reduced likelihood of symptomatic disease post-vaccination [16,17,18,19,20]. In addition to humoral responses, T cells have also been shown to play a vital role in the control of COVID-19 disease, including as long-term memory for the prevention of future episodes of severe disease [21,22]. In patients hospitalized for COVID-19, T cell levels have been correlated with disease outcome, with higher levels of CD8 T cells strongly associated with positive treatment outcomes [23]. In addition to amplifying antibody responses, sa-mRNA vaccines are expected to enhance T cell responses compared to conventional vaccines by more closely mimicking a natural viral infection [24]. Data from murine models of ARCT-021, another candidate sa-mRNA COVID-19 vaccine encoding the S glycoprotein of the original Wuhan-Hu-1 strain, support this theory, with higher proportions of interferon-γ producing CD8+ and CD4+ T cells across all tested doses of the sa-mRNA vaccine compared with conventional mRNA controls [25]. Given the commonalities between ARCT-154 and ARCT-021, it is highly likely that ARCT-154 will elicit a similar T cell profile, thus enhancing cellular immunity versus conventional mRNA vaccines, contributing to the increased efficacy we observed in this study. However, further research is needed to fully elucidate the effects of cellular immunity on ARCT-154 efficacy.

The results of this study are in line with those of Cromer et al. 2024 [26], who noted that vaccines with updated antigens elicit a 1.4-fold greater neutralizing antibody titer compared with historical vaccines. In contrast to BNT162b2, which is based on the original SARS-CoV-2 spike protein, ARCT-154 encodes the D614G variant, a mutation that quickly became dominant, and remains present in nearly all Omicron subvariants [27,28]. This difference may help to explain the differences in GMTs, and thus in rVE, between the two vaccines and provide insight into potential future protection against emerging SARS-CoV-2 variants. Based on conventional mRNA vaccines, Cromer et al. estimated that a 40% boost in antibody titers from an updated vaccine would translate to an rVE of 19% against symptomatic disease and 30% against severe disease compared to the historical vaccine, which is broadly in line with the estimates from our study, based on the stricter definitions of severe disease [26]. Although predicting the impacts on future circulating strains is purely speculative, a vaccine that generally induces higher SARS-CoV-2-specific neutralizing antibody titers would be expected to provide better protection than a vaccine which induces lower titers. In addition, ARCT-154 shows a greater breadth and durability of response through six months post-vaccination, particularly against Omicron BA.4/5 in older adults. Given the self-amplifying nature of ARCT-154, it would therefore be likely that this type of vaccine would also provide enhanced protection against future circulating strains compared with conventional mRNA vaccine technology and may be of particular benefit to older adults who remain at increased risk of severe COVID-19 outcomes.

As with all modelling studies, our analysis has a number of limitations. One of the major limitations was the lack of convalescent sera comparator in the phase 3 study of ARCT-154, which meant that we had to assume the ratio of BNT162b2-induced titer to convalescent sera associated with 50% protection from disease was the same as that observed in the previous clinical trial used as part of the model by Khoury et al. [13]. Although we acknowledge that this is a major assumption, Khoury et al. noted that despite the differences in study designs, case definitions, study timeframes, and definitions of convalescent individuals, there was a very high correlation between mean neutralization level and vaccine efficacy across the seven different vaccine studies (Spearman r = 0.905) [13]. Given this finding, the similarities found by Khoury et al. [13] between their estimates and observed efficacy studies, and the corresponding estimates for the association between efficacy and three different antibody markers (neutralizing antibodies, anti-spike IgG, and anti-receptor binding domain antibodies) in a separate phase 3 study of NVX-CoV2373 protein subunit vaccine [29], we felt justified in using the ratio of BNT162b2 GMT to convalescent sera derived from the Khoury et al. [13] study to estimate a theoretical ratio for comparison with ARCT-154. Although the absolute values for vaccine efficacy in our study may be potentially under- or over-estimated, this effect would be expected to be similar for each vaccine, thereby not affecting the conclusions regarding rVE. Another limitation of the phase 3 study was the relatively small sample size of participants ≥60 years of age. However, despite only having 33 and 35 participants in each vaccine group, respectively, by day 181 of the study, the 95% confidence intervals of the vaccine efficacy estimates against symptomatic disease caused by both Wuhan-Hu-1 and Omicron BA.4/5 did not overlap, indicating a clear difference in efficacy between the vaccines, reflected in the rVE estimates of 11% and 48%, respectively. Finally, this analysis was also limited by the vaccine immunogenicity data available for ARCT-154. Since the phase 3 study was initiated, other Omicron subvariants have become predominant; therefore, evaluating the rVE against Omicron BA.4/5 is less relevant now than at a time when these were the predominant circulating variants. Nevertheless, the results of this study demonstrate the increased breadth of protection offered by ARCT-154 compared to BNT162b2. Although future research is needed, it is likely that this will translate to improved efficacy against the current predominant circulating Omicron subvariants.

## 5. Conclusions

Our analysis has modelled the potential incremental benefits in vaccine efficacy of ARCT-154 versus BNT162b2 against both symptomatic and severe disease, with the highest differences observed in adults ≥60 years against the Omicron BA.4/5 subvariant. The results of this study translate the non-inferior immunogenicity against Wuhan-Hu-1 and superior immunogenicity against Omicron BA.4/5 seen in the phase 3 study into estimates of potential differences in efficacy, thereby demonstrating the enhanced potential of sa-mRNA vaccines to provide a more durable and broader response against COVID-19 than conventional mRNA vaccines, particularly in older adults who remain at increased risk of severe disease outcomes.

## Figures and Tables

**Table 1:** Geometric mean titers (95% confidence intervals) and ratios of geometric mean titers of neutralizing antibodies on days 1, 29, 91, and 181 for ARCT-154 vs BNT162b2 against SARS-CoV-2 Wuhan-Hu-1 and Omicron BA.4/5 by age group, per-protocol set.

Day	ARCT-154 *N* = 385	BNT162b2 *N* = 374	GMT Ratio ARCT-154 vs. BNT162b2
**Age < 60 years**			
***Wuhan-Hu-1***			
1	828 (724, 948) *n* = 352	886 (767, 1022) *n* = 339	0.93
29	5461 (4937, 6040) *n* = 345	3782 (3469, 4125) *n* = 333	1.44
91	5970 (5425, 6569) *n* = 337	2933 (2666, 3226) *n* = 325	2.04
181	4135 (3717, 4600) *n* = 302	1880 (1676, 2109) *n* = 287	2.20
***Omicron BA.4/5***			
1	281 (229, 344) *n* = 352	302 (242, 377) *n* = 339	0.93
29	2171 (1874, 2515) *n* = 345	1656 (1435, 1912) *n* = 333	1.31
91	1894 (1637, 2191) *n* = 337	905 (773, 1060) *n* = 325	2.09
181	1105 (942, 1295) *n* = 302	508 (419, 615) *n* = 287	2.18
**Age ≥ 60 years**			
***Wuhan-Hu-1***			
1	668 (450, 992) *n* = 33	695 (430, 1123) *n* = 35	0.96
29	4708 (3478, 6372) *n* = 33	3332 (2510, 4424) *n* = 34	1.41
91	5506 (4091, 7409) *n* = 32	2573 (1881, 3519) *n* = 31	2.14
181	3967 (2818, 5555) *n* = 30	1665 (1092, 2538) *n* = 26	2.38
***Omicron BA.4/5***			
1	226 (117, 438) *n* = 33	210 (107, 412) *n* = 35	1.08
29	1702 (928, 3121) *n* = 33	1336 (886, 2013) *n* = 34	1.27
91	1867 (1124, 3104) *n* = 32	721 (443, 1173) *n* = 31	2.59
181	1276 (704, 2313) *n* = 30	378 (200, 716) *n* = 26	3.38

GMT, geometric mean titer.

## Data Availability

The data presented in this study are available on reasonable request from the corresponding author.
